# Next-Generation Sequencing in the Diagnosis of Patients with Bardet–Biedl Syndrome—New Variants and Relationship with Hyperglycemia and Insulin Resistance

**DOI:** 10.3390/genes11111283

**Published:** 2020-10-29

**Authors:** Krzysztof Jeziorny, Karolina Antosik, Paulina Jakiel, Wojciech Młynarski, Maciej Borowiec, Agnieszka Zmysłowska

**Affiliations:** 1 Department of Pediatrics, Diabetology, Endocrinology and Nephrology, Medical University of Lodz, 90-419 Lodz, Poland; krzysztof.jeziorny@umed.lodz.pl; 2 Department of Clinical Genetics, Medical University of Lodz, 92-213 Lodz, Poland; karolina.antosik@umed.lodz.pl (K.A.); paulina.mludzik@umed.lodz.pl (P.J.); maciej.borowiec@umed.lodz.pl (M.B.); 3 Department of Pediatrics, Oncology and Hematology, Medical University of Lodz, 90-419 Lodz, Poland; wojciech.mlynarski@umed.lodz.pl

**Keywords:** Bardet–Biedl syndrome, NGS method, obesity, hyperglycemia, insulin resistance

## Abstract

Bardet-Biedl syndrome (BBS) is a rare autosomal recessively inherited disease with major clinical symptoms such as: obesity, retinal degeneration, polydactyly and renal abnormalities. The aim of the study was to assess the spectrum of gene variants among patients with BBS, identified on the basis of nationwide genetic studies of monogenic diabetes in Polish population. Out of 575 patients enrolled for genetic testing from February 2017 to July 2019, 25 patients with a clinical suspicion of BBS were selected. The identification of pathogenic variants was performed by using targeted next-generation sequencing (NGS) on Illumina NextSeq 550 platform involving the SureSelect assay (Agilent, Santa Clara, CA, USA). BBS was genetically confirmed in 10 of 25 suspected patients. In patients, 14 different variants were found in six genes, mainly in *BBS9* and *BBS10* gene, including two novel variants. A strong association between hyperglycemia and insulin resistance in patients and the presence of variants in *BBS9* gene was observed. Identification of 14 variants, including two new mutations using the NGS method, is the first molecular characteristic of Polish patients with Bardet–Biedl syndrome. It gives hope for earlier proper diagnosis of BBS in future patients selected from children with early childhood obesity and their medical multidisciplinary care.

## 1. Introduction

Bardet–Biedl syndrome (BBS) is a rare autosomal recessively inherited disease with the incidence of 0.7 cases per 100,000 [1,2,3]. The estimated prevalence of BBS is about 1:160,000 in northern European populations and 1:13,500 in selected Arab populations [1]. The main clinical symptoms of the syndrome include: early childhood obesity, retinal degeneration, polydactyly, hypogonadism, renal abnormalities and mental retardation. Other minor disorders diagnosed in the BBS patients are: type 2 diabetes mellitus (T2DM), cardiovascular problems, liver diseases, hypothyroidism and dental development abnormalities. The proposed clinical diagnosis of BBS is based on the presence of four major symptoms or three major and two minor ones [4].

BBS is a highly heterogenic disease. Not all clinical symptoms of BBS must occur in individual patients, even if pathogenic variants are present in the same gene. The appearance of some features may be related to the age of the patient, which may delay correct diagnosis. Even in patients in whom the same gene mutations have been confirmed, as well as within the same families with diagnosed BBS, the degree of expression of individual phenotypic features may vary [5,6].

The precise pathological mechanism of abnormalities observed in BBS is still unknown. Currently, it is believed that they are based on mutations in the genes encoding the proteins forming the BBSome and BBS chaperone complex responsible for the proper functioning of cilia and thus for the correct action of the signal pathways of body cells. BBS1, BBS2, BBS4, BBS5, BBS7, BBS8 and BBS9 proteins form the BBSome complex, and the chaperonin complex was created by BBS6, BBS10 and BBS12. The BBSome complex plays an important role in molecular and vesicular transport in and out of the primary cilium [1,7].

The diagnostic difficulties in the identification of the BBS patients are related not only to the number of pathogenic variants in many genes, which have so far been discovered in more than 20 [8] and the postulated model of triallelic inheritance [9], but also to overlapping clinical features of the disease with other ciliopathies with early obesity, such as Alstrom syndrome [10]. Therefore, further studies seem to be important for understanding the causes of the disease and thus to make an earlier accurate diagnosis and propose not only symptomatic but also causal treatment, including gene therapy [8].

The aim of the study was to evaluate the spectrum of gene variants and genotype–phenotype relationships among patients with clinical suspicion of Bardet–Biedl syndrome identified on the basis of nationwide genetic studies of monogenic diabetes in the Polish population.

## 2. Materials and Methods

The study protocol was approved by the University Bioethics Committee at the Medical University in Lodz, Poland (RNN/124/11/KE and RNN/343/17/KE). Patients and/or their parents gave written informed consent for participation in the study.

The patients with a clinical suspicion of BBS (based at least on obesity and visual disorders) were selected from the group of patients enrolled for genetic testing at the Outpatient Genetics Clinic of the Centre for Monogenic Diabetes in Lodz, Poland from February 2017 to July 2019.

**Figure 1:**
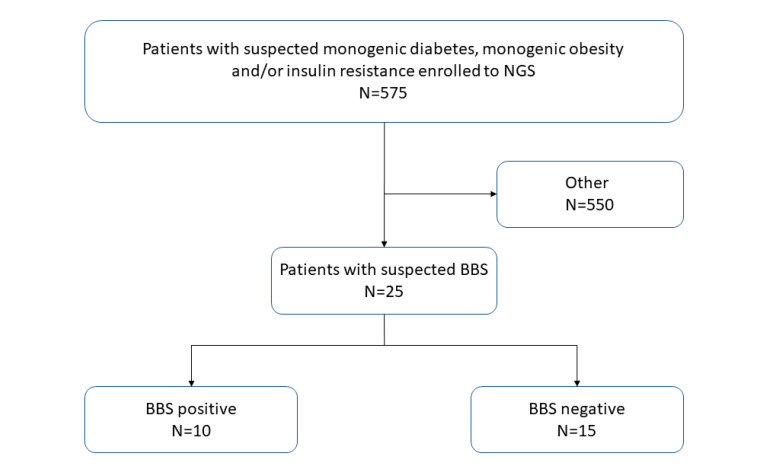
Pathway used on the process of patient recruitment for the present study. NGS—next-generation sequencing; BBS—Bardet–Biedl syndrome. N—sample size.

The study group consisted of 575 patients referred to the Centre for Monogenic Diabetes with suspected monogenic diabetes, monogenic obesity and/or syndromic insulin resistance aged from 3 months to 42 years and included 25 patients with suspected BBS based on obesity and visual disorders (Figure 1). First, each patient had a genetic consultation carried out by an experienced clinical geneticist in the Outpatient Genetics Clinic. Then, through the Genetics Clinic of the Monogenic Diabetes Center, he was directed to genetic analysis including a dedicated gene panel. After the molecular analysis confirming the diagnosis of BBS, the patient was referred to hospitalization, during which the severity of the disease was assessed. This analysis included metabolic tests (lipids profile, OGTT, HbA1c, levels of hormones and bone metabolites, insulin resistance based on HOMA-IR–homeostatic model of insulin resistance assessment) and ophthalmological, laryngological, psychological and dietary consultations.

Only one patient was qualified for genetic testing without obesity and visual impairment, but he was another patient’s younger brother and showed prenatal signs of polydactyly and kidney abnormalities.

Clinical data of each patient were collected from clinical records and a standardized questionnaire conducted during medical visits and then analyzed by an experienced clinician. Hyperglycemia or diabetes mellitus was diagnosed according to the WHO (World Health Organization) definition. BMI (body mass index) was defined as body weight divided by the square of body height (kg/m^2^). Then, the Z-score for the body mass index standard deviation score (BMI SDS) was calculated by LMS (least mean squares) method according to Cole et al. [11]. Renal impairment, including renal failure, renal cysts and other kidney defects were analyzed based on the clinical data obtained from ultrasounds and e-GFR (estimated glomerular filtration) levels. Learning problems were assessed on the basis of psychological consultation.

Twenty five patients including two individuals with pre-identified pathogenic variants of the BBS genes that were previously found in cooperation within the EURO-WABB project [3], qualified for re-analysis due to the much wider panel of BBS-related genes currently available. The genetic study was carried out at the Department of Clinical Genetics of the Medical University of Lodz, Poland.

DNA was isolated from peripheral blood cells (drawn into EDTA-coated vials) using automated Maxwell system (Promega, Madison, WI, USA) according to the manufacturer’s protocol. The identification of pathogenic variants was performed by using targeted next-generation sequencing (NGS) on Illumina NextSeq 550 platform involving the SureSelect assay (Agilent, Santa Clara, CA, USA). A custom panel was designed using SureDesign platform (www.earray.chem.agilent.com/suredesign/) and included 208 genes that are associated with monogenic diabetes and insulin resistance. Among them, expression of 23 genes related to BBS was evaluated (*ARL6, BBIP1, BBS1, BBS2, BBS4, BBS5, BBS7, BBS9, BBS10, BBS12, C8ORF37, CCDC28B, CEP290, IFT27, IFT74, LZTFL1, MKKS (BBS6), MKS1, SDCCAG8, TMEM67, TRIM32, TTC8 (BBS8), WDPCP*). Libraries were prepared with the Agilent Bravo Automated Liquid Handling Platform (Agilent, Santa Clara, CA, USA) according to the manufacturer’s protocol. NGS data processing and variant calling was performed in the Illumina BaseSpace (Illumina, San Diego, CA, USA). Variant annotation and filtering were carried out with the Illumina Variant Studio Software (Illumina, San Diego, CA, USA). Variants were classified according to the American College of Medical Genetics and Genomics recommendations [12]. In addition, we used VarSome.com The Human Genomics Community tool to assess the pathogenicity of the found variants (www.varsome.com) [13]. All pathogenic and likely pathogenic variants detected by NGS study were subsequently validated by the Sanger sequencing using an Applied Biosystems 3500 Genetic Analyzer (Applied Biosystems, Foster City, CA, USA) and Sequencher 5.0 software (Gene Codes Corporation, Ann Arbor, MI, USA).

### Statistical Analysis

Verification of the normality of distribution was carried out using the Shapiro–Wilk test. Categorical variables were presented as numbers with appropriate percentages and means with standard deviations (SD) and continuous variables as medians with interquartile range (25–75%). A heat map was created to connect selected clinical features in patients with variants found in BBS genes Analyses were performed using Statistica 13.1 PL software (Statsoft, Tulsa, OK, USA).

## 3. Results

BBS was genetically confirmed in 10 of 25 suspected patients. In 9 out of 10 BBS patients, obesity (BMI; mean ± SD: 27.2 ± 4.8 kg/m^2^, median 29.3 kg/m^2^ (25–75%: 22.4–31.1) and visual disturbances such as: cone–rod dystrophy, photophobia, nystagmus or cataract were observed. The other most frequent abnormalities were: polydactyly (in 8/10 of patients), renal impairment (in 7/10 of patients), insulin resistance (in 7/10 of patients), learning difficulties (in 7/10 of patients), dyslipidemia (in 6/10 of patients), cardiovascular problems (such as arterial hypertension, cardiomyopathy or valve defects) (in 5/10 of patients) and hearing loss (in 4/10 of patients). Moreover, in one of the patients, hypogonadotropic hypogonadism, and in another, bronchial asthma were diagnosed. Detailed clinical characteristics of patients are summarized in Table 1.

**Figure 2:**
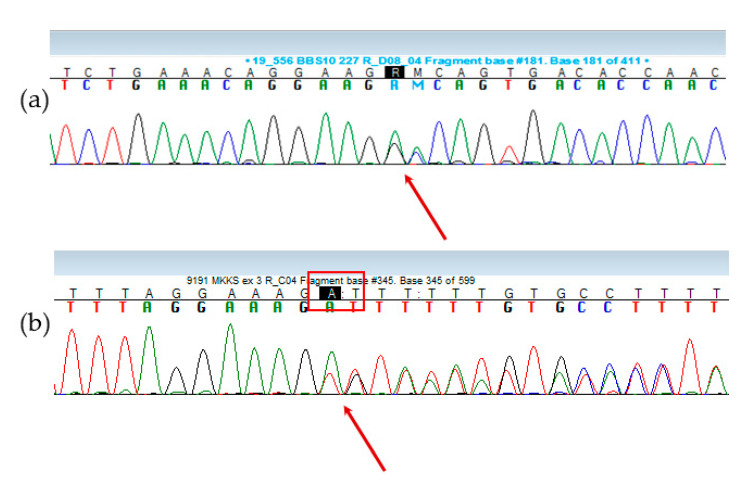
Chromatograms of the new variants: (**a**) NM_024685.4:c.680_681delGCinsAA in *BBS10* gene (Patient BBS No.1). The arrow indicates the location of the nucleotide change: two GC nucleotides are deleted and two A nucleotides are inserted: GGC (Gly) to GAA (Glu). (**b**) NM_170784.2:c.595_596delAG in *BBS6* gene (Patient BBS No.8 and BBS No.9). The arrow indicates the location of the nucleotide change: deletion of AG.

The mean age at genetic diagnosis of BBS was 10.8 ± 9.6 years. The median for age of genetic diagnosis was 8.1 years (25–75%: 3.9–15.5). The youngest patient was 4 months old at the time of genetic analysis. Molecular analysis in the study group revealed 14 different variants in six genes: *BBS2, BBS6* (*MKKS*), *BBS7, BBS8, BBS9* and *BBS10*. Two of them (NM_170784.2:c.595_596delAG in *BBS6* and NM_024685.4:c.680_681delGCinsAA in *BBS10*) are novel (Table 2 and Figure 2).

Analyzing the pathogenicity of two newly found variants, variant NM_170784.2:c.595_596delAG was classified as pathogenic, while variant NM_024685.4:c.680_681delGCinsAA was classified as likely pathogenic when it is detected in the case of autosomal recessive disease in trans with the pathogenic variant, which concerns BBS patient No.1.

Of the previously described variants presented in our BBS patients (BBS No.1–9), all are pathogenic or likely pathogenic. However, in BBS No.10, three variants were found, two of which in *BBS2* and *BBS8* genes were classified as VUS (variant of uncertain significance) and one variant in *BBS10* gene was described as likely benign, but potentially causative for Bardet–Biedl syndrome [14]. Interestingly, in asymptomatic parents of this child two variants were found in each of them, while in his father there was one new variant (VUS), which the patient did not have (Table 2). Moreover, genotyping of 17 other unaffected first-degree relatives of BBS patients confirmed the presence of the respective heterozygous variants in all individuals—15 parents (Table 2) and two siblings (BBS patient siblings No.2 and No.4).

Variants found in the study group were most often observed the *BBS9* and *BBS10* genes (3 patients each), *BBS6* and *BBS8* (2 patients each), *BBS2* and *BBS7* (1 patient each). Table 3 shows the association between selected clinical features in patients and variants found in *BBS* genes. Patient BBS No. 9 was excluded from this analysis because of his very young age at the time of genetic analysis (4 months of life) and due to the lack of other clinical features of BBS syndrome (different phenotype from his brother’s with the same variants in the *BBS6* gene). However, in addition to the apparent relationship between the majority of gene variants and the main clinical features of BBS, a strong association between hyperglycemia and insulin resistance and the presence of *BBS9* gene variants was observed (Table 3). Furthermore, looking at one of the patients (BBS No. 10), with three variants detected in three different genes (*BBS2*, *BBS8*, *BBS10*), in addition to obesity, renal impairment, hearing loss, learning disabilities and liver fibrosis, diabetes mellitus was also diagnosed.

## 4. Discussion

For the first time, using the NGS method, the analysis of the occurrence of genetic causative variants in the population of Polish patients with clinical suspicion of Bardet–Biedl syndrome was carried out and the genotype–phenotype correlations were assessed. Our research made it possible to find 14 mainly pathogenic/likely pathogenic variants in 10 patients and confirmed that the most common causative variants found in Polish BBS patients are located in *BBS9* and *BBS10* genes. Moreover, two of them (NM_170784.2:c.595_596delAG in *BBS6* and NM_024685.4: c.680_681delGCinsAA in *BBS10)* have not yet been described.

Other authors pointed to about 25% of the presence of causative mutations in the *BBS1* gene, whereas 20% concerned the *BBS10* gene, while the mutations in the other genes were less frequently observed in BBS patients [1]. However, in our study, identification of 4 out of 14 variants in both the *BBS9* and *BBS10* genes indicates almost 30% contribution of each of them to the genotype of Polish BBS patients.

A characteristic feature of BBS syndrome is its high phenotypic variability, which also occurs in patients with the same *BBS* gene mutations, even among family members [1,6]. It is known that the most common symptoms of BBS are visual abnormalities mainly related to retinal degeneration found in over 90% of patients, obesity in 72–92% and polydactyly in 63–81% of patients [1,4,6,15]. Our study showed similar observations of these phenotypic features present in 80–90% of patients. According to recent studies, renal disorders and mental retardation affect about half of BBS patients (20–53% and 50–61%, respectively) [6], compared to 70% of each of them in Polish BBS patients. Other accompanying symptoms seem to be much rarer in patients with BBS, with diabetes mellitus diagnosed in less than half of them [1,6]. In our study, hyperglycemia in the form of impaired glucose tolerance or diabetes mellitus was observed in 40% of patients and insulin resistance in 70% of the individuals.

Despite an increasing number of studies assessing the relations between the presented phenotype and genotype in BBS patients, it is difficult to find exact correlations and only trends in their presence are observed [6,16,17,18,19,20,21]. However, determination of direct correlations would enable early diagnosis, forecasting the occurrence of BBS complications and proposing prevention and dedicated treatment of coexisting symptoms. According to Deveault et al., the phenotype associated with the presence of causative variants in the *BBS10* gene is more severe than the presence of mutations in the *BBS1* or *BBS12* genes [19]. Interestingly, in our study we considered a patient with a triallelic inheritance including one variant in the *BBS10* gene, in whom we found numerous clinical symptoms and additionally T2DM. Among these various symptoms, the boy suffered from renal impairment, which is described as the most strongly associated with variants in the *BBS10* gene [17,18]. Moreover, in other patients from the study group, a relationship between mutations in the *BBS10* gene and the occurrence of renal impairment was also observed (seven patients with renal failure, three of them with variants in the *BBS10* gene). It should be emphasized that early detection of renal abnormalities in patients with BBS is extremely important because of the observed reduction of glomerular filtration, often leading to end-stage renal insufficiency. Renal malfunction is considered to be the main factor of morbidity and mortality among BBS patients [17].

In our study, a strong correlation between hyperglycemia and insulin resistance and *BBS9* gene variants was noted. So far, there was no clear association between the presence of insulin resistance or hyperglycemia/diabetes and genotype in BBS patients. Deveault et al. noticed that the large deletion of the *BBS9* gene can lead to the development of a more severe BBS phenotype (e.g., osteopenia with pathological fractures leading to disability, kidney and liver failure, behavioral disorders) and the development of T2DM [19]. In addition, some studies have observed lower insulin resistance measured by HOMA-IR and lower abdominal fat index for variants of the *BBS1* gene compared to the *BBS10* gene [14,16]. However, patients with BBS have higher fasting glycaemia and insulin resistance [14,16]. It is also believed that the development of T2DM or insulin resistance in BBS patients may be related to obesity and other components of metabolic syndrome [1].

The limitation of the research is the small size of the target study group. However, this seems understandable given that BBS is a very rare genetic disease. It is also worth noting that our analysis of genotype–phenotype correlation did not take into account the youngest patient in our population, and perhaps even in the world, diagnosed in the 4th month of life, who did not yet show any accompanying symptoms. We are not sure whether this is due only to his age or individual differences within the same family. Additionally, in a patient BBS No.10, with the presence of three variants in three different genes, it is difficult to clearly indicate without functional tests whether all or which of these three mutations are causative for the phenotype presented.

## 5. Conclusions

In conclusion, clinical and genetic heterogeneity of patients with Bardet–Biedl syndrome is an important diagnostic challenge. The use of modern molecular analysis techniques, including NGS method, enables early and proper genetic confirmation of BBS syndrome and broadening the spectrum of known causative variants. Due to the increased morbidity and mortality in this group of patients it seems important to diagnose the disease as quickly and precisely as possible and disclose all phenotype-genotype correlations, which will allow to provide patients with Bardet–Biedl syndrome with early multi-specialist care. This will enable preventive interventions and symptomatic treatment of patients, including hyperglycemia and insulin resistance.

Therefore, our efforts are aimed at drawing attention to rare diseases such as Bardet–Biedl syndrome and increasing awareness among the widest possible range of clinicians as well as scientists dealing with molecular genetics and biotechnology. Only our close cooperation in the search for clinical symptoms in patients by doctors from various fields of medicine and early referral to molecular analysis will allow BBS patients to receive appropriate care.

## Figures and Tables

**Table 1:** Detailed clinical characteristics of the patients with Bardet–Biedl syndrome.

Patient ID	Gene	Sex (F/M)	Age at Genetic Diagnosis (Years)	BMI Z-Score	Ophthalmologic Abnormalities	Polydactyly	Obesity	Learning Problems	Renal Impairment	Insulin Resistance	Hyperglycemia/Diabetes Mellitus (DM)	Cardiovascular Problems	Hearing Loss	Other Manifestation
BBS No. 1	*BBS10*	M	15.5	2.3	Yes	Yes	Yes	No	Yes	Yes	No	Yes	No	bronchial asthma, cryptorchidism, dyslipidemia, scoliosis
BBS No. 2	*BBS9*	M	33.0	2.5	Yes	Yes	Yes	Yes	No	Yes	IGT	Yes	Yes	-
BBS No. 3	*BBS8*	M	5.7	3.1	Yes	Yes	Yes	Yes	Yes	Yes	No	Yes	Yes	dyslipidemia, autism
BBS No. 4	*BBS9*	F	2.8	4.2	Yes	Yes	Yes	Yes	Yes	Yes	IGT	No	No	dyslipidemia
BBS No. 5	*BBS10*	M	12.8	2.5	Yes	Yes	Yes	No	Yes	No	No	Yes	No	dyslipidemia, hypospadias
BBS No. 6	*BBS9*	F	10.2	3.1	Yes	No	Yes	Yes	No	Yes	IGT	No	No	dyslipidemia
BBS No. 7	*BBS7*	F	3.9	4.5	Yes	Yes	Yes	Yes	No	Yes	No	No	No	-
BBS No. 8 (brother)	*BBS6*	M	6.0	3.4	Yes	Yes	Yes	Yes	Yes	No	No	Yes	Yes	hypogonadism
BBS No. 9 (brother)	*BBS6*	M	0.3	1.0	No	Yes	No	No	Yes	No	No	No	No	-
BBS No. 10	*BBS2* *BBS8* *BBS10*	M	17.4	2.0	No	No	Yes	Yes	Yes	Yes	DM	No	Yes	hepatic steatosis, dyslipidemia, scoliosis

IGT—impaired glucose tolerance; DM—diabetes mellitus; BMI—body mass index.

**Table 2:** Summary of variants detected in Polish Bardet–Biedl syndrome cohort.

Patient ID	Gene	Nucleotide Change	Protein Change	Genotype of Patient	Variant from Mother	Variant from Father
BBS No. 1	*BBS10*	NM_024685.4:c.145C>T	NP_078961.3:p.Arg49Trp	Compound heterozygous	NM_024685.4:c.145C>T Heterozygous	NM_024685.4:c.680_681delGCinsAA Heterozygous
		**NM_024685.4:c.680_681delGCinsAA**	**NP_078961.3:p.Gly227Glu**			
BBS No. 2	*BBS9*	NM_198428.3:c.1693+1G>A	-	Compound heterozygous	NM_198428.3:c.1693+1G>A Heterozygous	NM_198428.3:c.190C>T Heterozygous
		NM_198428.3:c.190C>T	NP_940820.1:p.Gln64Ter			
BBS No. 3	*BBS8*	NM_144596.3:c.489G>A	NP_653197.2:p.Thr163=	Homozygous	NM_144596.3:c.489G>A Heterozygous	N/A
BBS No. 4	*BBS9*	NM_198428.3:c.1789+1G>C	-	Compound heterozygous	NM_198428.3:c.1789+1G>C Heterozygous	NM_198428.3:c.190C>T Heterozygous
		NM_198428.3:c.190C>T	NP_940820.1:p.Gln64Ter			
BBS No. 5	*BBS10*	NM_024685.4:c.145C>T	NP_078961.3:p.Arg49Trp	Compound heterozygous	NM_024685.4:c.271dupT Heterozygous	NM_024685.4:c.145C>T Heterozygous
		NM_024685.4:c.271dupT	NP_078961.3:p.Cys91LeufsTer5			
BBS No. 6	*BBS9*	NM_198428.3:c.1540C>T	NP_940820.1:p.Arg514Ter	Homozygous	NM_198428.3:c.1540C>T Heterozygous	NM_198428.3:c.1540C>T Heterozygous
BBS No. 7	*BBS7*	NM_176824.3:c.1968delA	NP_789794.1:p.Gln657ArgfsTer17	Homozygous	NM_176824.3:c.1968delA Heterozygous	NM_176824.3:c.1968delA Heterozygous
BBS No. 8 (brother)	*BBS6*	**NM_170784.2:c.595_596delAG**	**NP_740754.1:p.Ser199PhefsTer22**	Compound heterozygous	NM_170784.2:c.1436C>G Heterozygous	NM_170784.2:c.595_596delAG, Heterozygous
		NM_170784.2:c.1436C>G	NP_740754.1:p.Ser479Ter			
BBS No. 9 (brother)	*BBS6*	**NM_170784.2:c.595_596delAG**	**NP_740754.1:p.Ser199PhefsTer22**	Compound heterozygous		
		NM_170784.2:c.1436C>G	NP_740754.1:p.Ser479Ter			
BBS No. 10	*BBS2*	NM_031885.4:c.1381G>A	NP_114091.3:p.Val461Met	Triallelic	NM_031885.4:c.1381G>A Heterozygous	-
	*BBS8*	NM_144596.3:c.725G>A	NP_653197.2 p.Arg242His		NM_144596.3:c.725G>A Heterozygous	-
	*BBS10*	NM_024685.4:c.424G>A	NP_078961.3:p.Asp142Asn		-	NM_024685.4:c.424G>A Heterozygous NM_024685.4:c.411G>C Heterozygous

#Numbering of nucleotides: +1=A of ATG codon No entry in the column protein change means splice donor variant. Novel mutations indicated in bold. N/A—not available.

**Table 3:** The association (the heat map) between selected clinical features in patients and variants found in *BBS* genes.

	Gene	*BBS2*	*BBS6*	*BBS7*	*BBS8*	*BBS9*	*BBS10*
Phenotype							
**Ophtalmologic abnormalities**		0% (0/1)	100% **(1/1)**	100% **(1/1)**	50% (1/2)	100% **(3/3)**	66% (2/3)
**Polydactyly**		0% (0/1)	100% **(1/1)**	100% **(1/1)**	50% (1/2)	66% (2/3)	66% (2/3)
**Obesity**		100% **(1/1)**	100% **(1/1)**	100% **(1/1)**	100% **(2/2)**	100% **(3/3)**	100% **(3/3)**
**Learning problems**		100% **(1/1)**	100% **(1/1)**	100% **(1/1)**	100% **(2/2)**	100% **(3/3)**	33% (1/3)
**Renal impairment**		100% **(1/1)**	100% **(1/1)**	0% (0/1)	100% **(2/2)**	33% (1/3)	100%
**Insulin resistance**		100% **(1/1)**	0% (0/1)	100% **(1/1)**	100% **(2/2)**	100% **(3/3)**	66% (2/3)
**Hyperglycemia**		0% (0/1)	0% (0/1)	0% (0/1)	0% (0/2)	100% **(3/3)**	0% (0/3)
**Diabetes mellitus**		100% **(1/1)**	0% (0/1)	0% (0/1)	50% (1/2)	0% (0/3)	33% (1/3)
**Cardiovascular problems**		0% (0/1)	100% **(1/1)**	0% (0/1)	50% (1/2)	33% (1/3)	66% (2/3)
**Hearing loss**		100% **(1/1)**	100% **(1/1)**	0% (0/1)	100% **(2/2)**	33% (1/3)	33% (1/3)

The youngest patient/BBS patient No. 9 was excluded from this analysis—details in text. Numbers in brackets indicate frequency of symptoms/number of specific gene variants. 100% indicated in bold.
